# Electrospun Poly(ethylene-*co*-vinyl alcohol)/Graphene Nanoplatelets Composites of Interest in Intelligent Food Packaging Applications

**DOI:** 10.3390/nano8100745

**Published:** 2018-09-20

**Authors:** Sergio Torres-Giner, Yolanda Echegoyen, Roberto Teruel-Juanes, Jose D. Badia, Amparo Ribes-Greus, Jose M. Lagaron

**Affiliations:** 1 Novel Materials and Nanotechnology Group, Institute of Agrochemistry and Food Technology (IATA), Spanish Council for Scientific Research (CSIC), Calle Catedrático Agustín Escardino Benlloch 7, 46980 Paterna, Spain; storresginer@iata.csic.es (S.T.-G.); yolanda.echegoyen@iata.csic.es (Y.E.); 2 Technological Institute of Materials (ITM), Universitat Politècnica de València (UPV), Camí de Vera s/n, 46022 Valencia, Spain; rotejua@upvnet.upv.es (R.T.-J.); aribes@ter.upv.es (A.R.-G.); 3 Department of Chemical Engineering, School of Engineering, Universitat de València, Camí de les Universitats s/n, 46800 Burjassot, Spain; jose.badia@uv.es

**Keywords:** EVOH, graphene, electrospinning, smart labels, intelligent packaging

## Abstract

Graphene nanoplatelets (GNPs) were synthetized from graphite powder and, thereafter, embedded in poly(ethylene-*co*-vinyl alcohol) (EVOH) fibers by electrospinning in the 0.1–2 wt.-% range. The morphological, chemical, and thermal characterization performed on the electrospun nanocomposite fibers mats revealed that the GNPs were efficiently dispersed and rolled along the EVOH fibrilar matrix up to contents of 0.5 wt.-%. Additionally, the dielectric behavior of the nanocomposite fibers was evaluated as a function of the frequency range and GNPs content. The obtained results indicated that their dielectric constant rapidly decreased with the frequency increase and only increased at low GNPs loadings while the nanocomposite fiber mats became electrically conductive, with the maximum at 0.5 wt.-% GNPs content. Finally, the electrospun mats were subjected to a thermal post-treatment and dark films with a high contact transparency were obtained, suggesting that the nanocomposites can be used either in a nonwoven fibers form or in a continuous film form. This study demonstrates the potential of electrospinning as a promising technology to produce GNPs-containing materials with high electrical conductivity that can be of potential interest in intelligent packaging applications as “smart” labels or tags.

## 1. Introduction

In the field of intelligent packaging, the use of electrically conductive polymer-based materials opens up new opportunities to create “smart” labels or tags. Intelligent or smart packaging is the umbrella term for a range of intelligent technologies that allow packaging to contain, evaluate, and transmit relevant information [1]. For instance, smart packages can enable monitoring of the conditions and quality of the packaged products (e.g., food freshness) from the production line to the end user. This includes relevant information and spoilage indicators such as time, temperature, and pH or the presence of different gases, chemical contaminants, pathogens, etc. Apart from this, smart labels can also include components that range from bar codes to radio frequency transmitters, i.e., radio frequency identification (RFID) devices and printed electronics [2]. The smart tags can be used to electronically transfer information from the packaging to the consumer about the packaged material through, for instance, refrigerator displays. Therefore, intelligent packaging systems can make packaging more informative and interactive whereas their global demand is expected to grow strongly to reach US $1.5 billion by 2025 [2].

Currently, however, the intelligent packaging technology habitually requires the use of a silicon chip as the substrate for the high-frequency electronics, limiting its application for packaging uses. In addition, there is an increasing necessity for the development of new thin-film transmitters that can be efficiently embedded in the packaging structure. In this sense, the use of poly(ethylene-*co*-vinyl alcohol) (EVOH) copolymers could represent an advantageous strategy since this family of copolymers is frequently employed in high-barrier packaging films in the form of inner layers with very low thickness (typically well below 10 μm). In addition, EVOH films are highly transparent and hydrophilic, yet water-insoluble. Therefore, EVOH is a suitable candidate to be efficiently employed for the inclusion of smart tags in the packaging structure and/or for the creation of patterns acting like a bar code. This would provide a unique response to electrical stimuli that give relevant information about the physicochemical properties of the foodstuff packaged and/or for traceability and improved supply chain management (SCM) purposes.

Graphene was the last carbon allotrope to be discovered, after fullerenes and carbon nanotubes (CNTs) [3]. This carbonaceous material is presented in the form of a unique two-dimensional (2D) macromolecular sheet of carbon atoms with a honeycomb-like structure, the so-called graphene nanoplatelets (GNPs), which has become one of the most promising materials available today. Compared to other nano-sized carbonaceous systems (e.g., CNTs), GNPs have attracted considerable attention because of their combination of outstanding mechanical flexibility, excellent electrical and thermal conductivity, optical transparency, and low density. The peculiar properties of single layers of graphene include a Young’s modulus of ~1 TPa, an electrical conductivity (σ) of approximately 6000 S/cm, a thermal conductivity (λ) of up to 5300 W/m·k, a surface area of over 2600 m^2^/g, a high chemical tolerance, and a broad electrochemical window [4,5]. As a result, GNPs have been widely considered as a perfect filler to develop novel carbon-based reinforced polymer nanocomposites with enhanced thermal, electrical, and mechanical properties [6,7]. In this sense, the use of GNPs-containing plastics is very advantageous for several applications in energy and electronics, but their use can also be originally focused on intelligent packaging strategies in combination to EVOH since it is a food contact polymer with excellent optical properties and polarity.

Electrohydrodynamic processing (EHDP) is a straightforward, versatile, and low-cost technique based on the application of high electrical fields to a viscoelastic polymer solution or melt via a metallic capillary orifice that allows to fabricate polymer nanostructures with different functionalities [8]. EHDP is habitually referred to as electrospinning when fiber-based morphologies are produced in which a wide range of polymers and biopolymers can be processed. Electrospun nanofibers can find several applications in the packaging industry, including the development of active and intelligent systems [9,10]. Although the electrospun materials are predominantly polymer based, certain amount of non-polymer contents (e.g., nano-sized fillers) can also be incorporated into the primary electrospinning solution to form hybrid ultrathin or nanocomposite fibers [11,12,13]. At present, the incorporation of GNPs has been achieved into electrospun fibers of poly(vinylpyrrolidone) (PVP) as a conductive additive to enhance the high-rate capabilities for lithium-ion batteries [4], polystyrene (PS) and polyvinyl chloride (PVC) to generate superhydrophobic surfaces [14], poly(vinyl acetate) (PVAC) to improve the optical absorption for ultrafast photonics [15], polyacrylonitrile (PAN) to produce carbon nanofibers (CNFs) [16], polyaniline/poly(methyl methacrylate) (PANi/PMMA) blends for conductive devices [17], and PAN/PVP blends as high capacitance materials [18].

In this work, for the first time, EVOH/GNPs nanofibers were prepared by electrospinning. The morphology, thermal properties, and electrical conductivity of the resultant hybrid nanofibers mats were characterized. Finally, the electrospun mats were subjected to a thermal post-treatment in order to generate films that could be applied for creating smart labels or tags with high electrical rate capabilities in the field of intelligent food packaging.

## 2. Experiment

### 2.1. Materials

EVOH containing 32 mol% ethylene content, i.e., EVOH32, was supplied by Nippon Gohsei (Osaka, Japan) as Soarnol™ DC3212B. The copolymer has a density of 1.19 g/m^3^ (23 °C) and a melt flow rate (MFR) of 12 g/10 min (210 °C, 2160 g). Graphite powder, G282863 grade, was purchased from Sigma-Aldrich S.A. (Madrid, Spain). Sodium nitrate (NaNO_3_), hydrogen peroxide solution (H_2_O_2_) at 30 wt.-%, hydrazine hydrate (N_2_H_4_) at 50–60 wt.-%, ammonia solution (NH_4_OH) at 25 wt.-%, potassium permanganate (KMnO_4_) of 97% purity, and isopropyl alcohol (IPA) with purity ≥99% were also purchased from by Sigma-Aldrich S.A. Sulfuric acid (H_2_SO_4_) of 96% purity was provided by Panreac S.A. (Barcelona, Spain).

### 2.2. Oxidation of Graphite

In a first stage, graphite oxide (GO) was prepared by oxidizing graphite powder based on the modification of the so-called Hummer’s method described by Hirata et al. [19]. Briefly, 10 g of graphite powder and 7.5 g of NaNO_3_ were placed into a 2000 mL round-bottom glass flask. Then, 621 g of H_2_SO_4_ was added and the mixture was stirred while being cooled in an ice water bath. Thus, 45 g of KMnO_4_ was gradually added for about 1 h. Cooling was completed after 2 h and the mixture was allowed to stand for five days at about 20 °C with gentle stirring to obtain a highly viscous liquid. After this, 1000 cm^3^ of 5 wt.-% H_2_SO_4_ aqueous solution was gradually added to the resultant solution for 1 h under continuous and gentle stirring. The resultant mixture was further stirred for 2 h. Then, 30 g of the H_2_O_2_ aqueous solution was added to the above liquid and the mixture was stirred for another 2 h. In order to remove the ions of oxidant origin, especially manganese ions, the resultant liquid was purified by repeating the following procedure cycle 15 times: Centrifugation, removal of the supernatant liquid, addition of a mixed aqueous solution of 3 wt.-% H_2_SO_4_/0.5 wt.-% H_2_O_2_, and shaking to re-disperse. The mixed solution amounted, in total, to about 13 g. The purification procedure was similarly repeated a further three times, except that the liquid to be added was replaced with water. The resultant mixture was allowed to stand for at least 24 h to precipitate thick particles, which were filtered and removed. The remaining dispersion was purified several times with water. Finally, a brown-black viscous flurry containing GO particles was obtained. The GO content was ca. 1 wt.-%, as determined from weight difference of the dispersion before and after drying at 150 °C for 1 h in an oven.

### 2.3. Reduction of Graphite Oxide

In a second stage, the resultant GO particles were reduced to graphene using NH_4_OH based on previous methodology [20]. For this, 0.1 g of the above-obtained GO particles was mixed with 100 mL of deionized water in a 250 mL round-bottom glass flask, yielding an inhomogeneous yellow-brown dispersion. This dispersion was bath ultrasonicated using a Fisher Scientific FS60 Ultrasonic Cleaner (150 W) from Thermo Fisher Scientific (Waltham, MA, USA) until it became clear, i.e., with no visible particulate matter. Then, 10 mL of N_2_H_4_ and 10 mL of NH_4_OH were added and the resultant solution was heated overnight in an oil bath at 90 °C under a water-cooled condenser, in which the reduced GO gradually precipitated out as a black solid. The final suspension was isolated by filtration and then vacuum-dried at 60 °C until the solids reached a concentration of ca. 0.1 wt.-%.

### 2.4. Preparation of Electrospun Fiber Mats

The polymer solution for electrospinning was prepared by fully dissolving 7 wt.-% EVOH in 4/1 (vol./vol.) IPA/water. The obtained EVOH solution was then added to the above-described water-based solution containing graphene, pre-heated at 50 °C, to reach the following weight contents of GNPs in EVOH: 0.1, 0.5, 1, and 2 wt.-%. The resultant GNPs dispersions in EVOH were then ultrasonicated for 15 min and transferred immediately to a 5 mL plastic syringe. A control solution of EVOH without GNPs was prepared in identical conditions.

The electrospinning process was performed using a Fluidnatek^®^ LE-50 benchtop line from Bioinicia S.L. (Valencia, Spain) with a dual polarizer yielding a variable high-voltage ranging from 0–60 kV and with temperature and humidity control. The EVOH solutions containing the GNPs were pumped through a stainless-steel needle injector and collected on a grounded metallic flat plate. The applied voltage, flow-rate, and tip-to-collector distance were set at 15 kV, 0.5 mL/h, and 15 cm, respectively. All samples were electrospun in a controlled environmental chamber at 29 °C and 30% relative humidity (RH). The electrospun fiber mat thickness was ca. 200 microns. All the characterization work was carried out in the electrospun fibers mats.

Additionally, the obtained electrospun fibers mats were proven to be conformable into continuous films by annealing in a hydraulic press 4122-model from Carver, Inc. (Wabash, IN, USA). This step was optimally performed at 158 °C, without pressure, for 10 s. The resultant films were air cooled at room temperature.

### 2.5. Morphology

The morphology of the electrospun mats was examined by scanning electron microscopy (SEM) with a S-4800 from Hitachi (Tokyo, Japan). Prior to examination, samples were deposited on suitable beveled microscopy holders and sputtered using a gold-palladium mixture under vacuum. All SEM experiments were carried out at 8.0 kV.

Transmission electron microscopy (TEM) was performed using a JEOL 1010 from JEOL USA, Inc. (Peabody, MA, USA) equipped with a digital image acquisition system from Bioscan (Edmonds, WA, USA). TEM images were taken directly on mats electrospun onto the TEM observation grids. At least 25 SEM and TEM micrographs were analyzed for each sample using Adobe Photoshop 7.0 software from Adobe Systems Incorporated (San Jose, CA, USA) to determine the sizes from their original magnification.

### 2.6. Wide Angle X-ray Scattering

Wide angle X-ray scattering (WAXS) was performed using a D5000 X-ray Powder Diffractometer from Siemens AG (Munich, Germany). Radial scans of intensity vs. scattering angle (2θ) were recorded at room temperature in the range 2 to 50° (2θ), step size of 0.03° (2θ), scanning rate of 8 s/step, with identical settings of the instrument by using a filtered Cu Kα radiation (λ = 1.5406 Å), an operating voltage of 40 kV, and a filament current of 30 mA. Bragg’s law (n·λ = 2·*d*·sinθ) was applied to calculate the basal spacing (*d*).

### 2.7. Raman Imaging

Raman images were taken with a Jasco NRS-3100 Confocal Micro-Raman spectrophotometer from Jasco Inc. (Easton, MD, USA) using a short working distance 100× objective, which according to the manufacturer provides a lateral and depth resolution of ca. 1 and 2 μm, respectively under high confocal conditions. The source was a red laser in the visible excitation tuned at 632.8 nm. Raman chemical images were carried out in the point by point mode by plotting the added area of the two graphene peaks present in the spectral 1780–918 cm^−1^ area and were constructed by taking 15 × 15 spectra equally spaced along a similar flat and continuous sample area in the case of the samples from 0.1 to 1 wt.-% GNPs loading and 20 × 20 spectra for the sample with 2 wt.-% GNPs content. Averaged spectra were taken in the samples with the 40× objective in a non-confocal mode.

### 2.8. Thermal Analysis

Thermal transitions of the electrospun EVOH nanofibers were evaluated by differential scanning calorimetry (DSC) using a DSC-7 analyzer from PerkinElmer, Inc. (Waltham, MA, USA) equipped with the refrigerating cooling accessory Intracooler 2. For this, ca. 2 mg samples were placed in 40-μL hermetic aluminum sealed pans, previously calibrated with an indium standard. The scanning rate was 10 °C/min. Samples were subjected to a first heating step from −25 to 200 °C, followed by a cooling step down to −25 °C, and a second heating step to 200 °C. An empty aluminum pan was used as a reference and all tests were carried out, at least, in triplicate.

### 2.9. Dielectrical Performance and Electrical Conductivity

The dielectric spectra of the samples were obtained using an alpha (α) mainframe frequency analyzer, in conjunction with an active cell Concept 40, from Novocontrol Technologies BmgH & Co. Kc (Hundsangen, Germany). The sample electrode assembly consisted of two stainless steel electrodes filled with the polymer. The diameters of the electrodes were 20 mm and the thickness values of each sample were determined for each measurement. A single-sweep experiment was performed. The spectra were measured in the frequency (*f*) range of 10^−2^–10^7^ Hz, under isothermal conditions at a temperature of 25 °C in nitrogen atmosphere. The analysis was conducted through the complex dielectric permeability ε* = ε′ − *i*·ε″, taking into account the real (ε′) and imaginary (ε″) parts as well as the loss tangent (tan δ = ε″/ε′). In order to discriminate polarization and conductive effects [21,22], the complex electric modulus (M*) was obtained as follows:

$$M^{*}=\frac{1}{\varepsilon^{*}}=\frac{1}{\varepsilon^{\prime }-i\cdot \varepsilon^{\prime \prime }}=\frac{\varepsilon^{\prime }}{{\varepsilon^{\prime }}^{2}+{\varepsilon^{\prime \prime }}^{2}}+i\cdot \frac{\varepsilon^{\prime \prime }}{{\varepsilon^{\prime }}^{2}+{\varepsilon^{\prime \prime }}^{2}}=M^{\prime }+i\cdot M^{\prime \prime }$$

where

$$M^{\prime }=\frac{\varepsilon^{\prime }}{{\varepsilon^{\prime }}^{2}+{\varepsilon^{\prime \prime }}^{2}}$$

$$M^{\prime \prime }=\frac{\varepsilon^{\prime \prime }}{{\varepsilon^{\prime }}^{2}+{\varepsilon^{\prime \prime }}^{2}}$$

The complex electrical conductivity σ* = ε*·ε_0_·ω was analyzed by means of the same experimental set. The values of the direct current electrical conductivity (σ_dc_) were calculated by extrapolating the conductivity plateau to *f*→0 [23].

## 3. Results and Discussion

### 3.1. Characterization of GNPs

**Figure 1:**
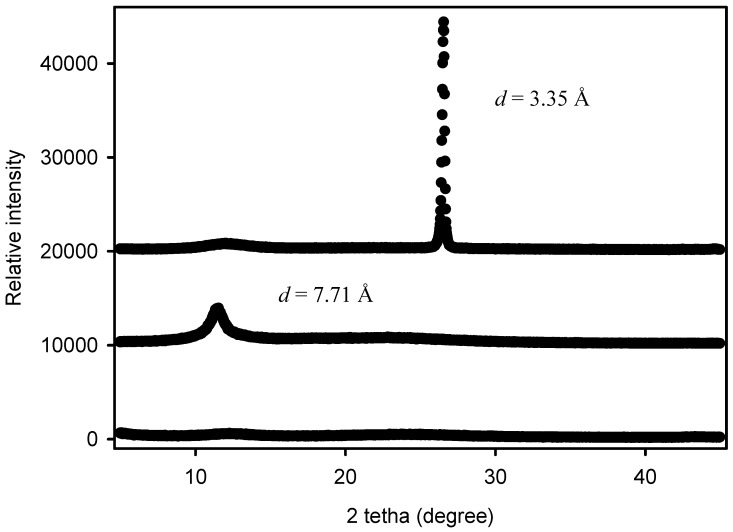
Wide angle X-ray scattering (WAXS) diffractograms of, from top to bottom, pristine graphite, graphite oxide (GO), and graphene nanoplatelets (GNPs). The interlayer distance is represented by *d*.

To confirm the detailed microstructure of the processed-graphite particles, the crystal phases of graphite, GO, and GNPs were analyzed by WAXS. From Figure 1 it can be observed that the XRD patterns of pristine graphite exhibits a strong peak centered at ~26.6° (2θ), which can be attributed to the typical graphite diffraction plane (002). By applying Bragg’s law, this resulted in a *d* value of 3.35 Å, which is the characteristic interlayer distance between graphite layers [24]. Instead, a weak and broad basal diffraction peak was observed in the X-ray diffraction pattern of GO at ~11.4° (2θ), which corresponds to a *d* value between planes of 7.71 Å. This significant spacing increase reveals that the oxygen-containing functional groups were intercalated in the interlayer of graphite, then confirming the oxidation of graphite to GO. Furthermore, the lower intensity of this characteristic peak indicates that the degree of crystallinity in the GO structure was reduced. Indeed, GO is known to consist of heavily oxidized graphene sheets, which are loosely attached to each other [11]. Finally, the absence of peaks in the WAXS spectrum of the GNPs suggests that the graphene tactoids were randomly stacked.

**Figure 2:**
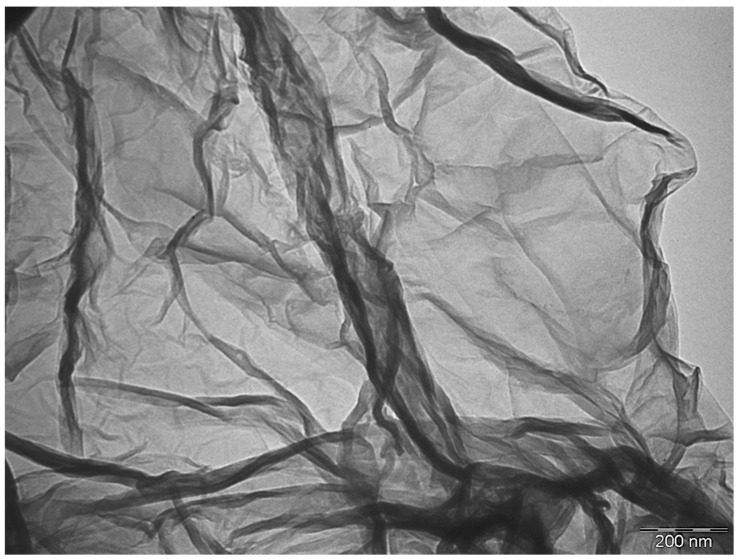
Transmission electron microscope (TEM) image of a single graphene nanoplatelet (GNP). Scale marker is 200 nm.

Figure 2 shows a representative image, obtained by TEM, of a single layer of graphene. This provides direct evidence for the existence of a flat-like structure with a large surface and a diameter in the nanometric range, the so-called nanoplatelet. Previous studies have well described the typical morphology of a graphene particle, describing that it forms a 2D structure based on a cluster composed by several monolayers in which typical sizes of individual particles can reach several microns [25].

### 3.2. Morphology of EVOH/GNPs Fibers

**Figure 3:**
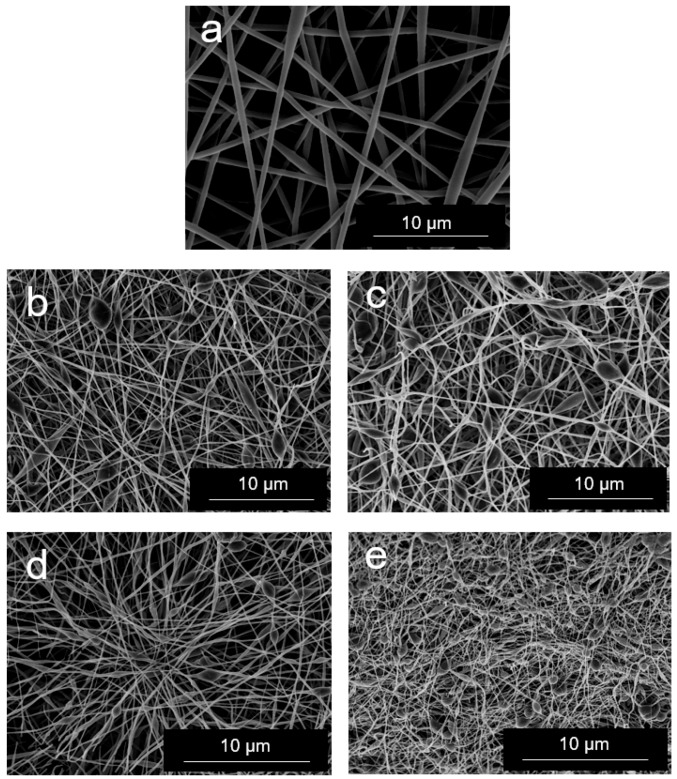
Scanning electron microscopy (SEM) images of electrospun fibers of: (**a**) Neat poly(ethylene-*co*-vinyl alcohol) (EVOH); (**b**) EVOH/graphene nanoplatelets (GNPs) at 0.1 wt.-%; (**c**) EVOH/GNPs at 0.5 wt.-%; (**d**) EVOH/GNPs at 1 wt.-%; (**e**) EVOH/GNPs at 2 w.-t%. Scale markers are 10 μm.

Figure 3 shows the SEM images of the electrospun EVOH and EVOH/GNPs fibers. From Figure 3a it can be seen that the neat EVOH fibers presented a fibrilar morphology, completely free of beaded regions, with a mean diameter of approximately 675 nm. The resultant morphology differs from that previously reported in the study performed by Martínez-Sanz et al. [26], in which EVOH fibers presented beaded regions, this being mainly related to the lower polymer concentration used in the solution for electrospinning. As shown in Figure 3b–e, increasing the GNPs content led to a significant decrease in the electrospun fibers diameter. In particular, the average diameter was reduced from approximately 425 nm, for the fibers containing 0.1 wt.-% GNPs, to a value below 100 nm, for those fibers containing 2 wt.-% GNPs. This diameter decrease of the electrospun EVOH fibers can be related to an increase in the solution conductivity when the GNPs were added. In addition to a decrease in the fibers diameter, the GNPs incorporation gave rise to the formation of certain beaded fibers. This effect was particularly notable at the highest GNPs content, i.e., 2 wt.-%, where the bead morphology was the most prevalent. This observation preliminary suggests that GNPs agglomeration could occur at high contents inside the EVOH fibers during electrospinning.

**Figure 4:**
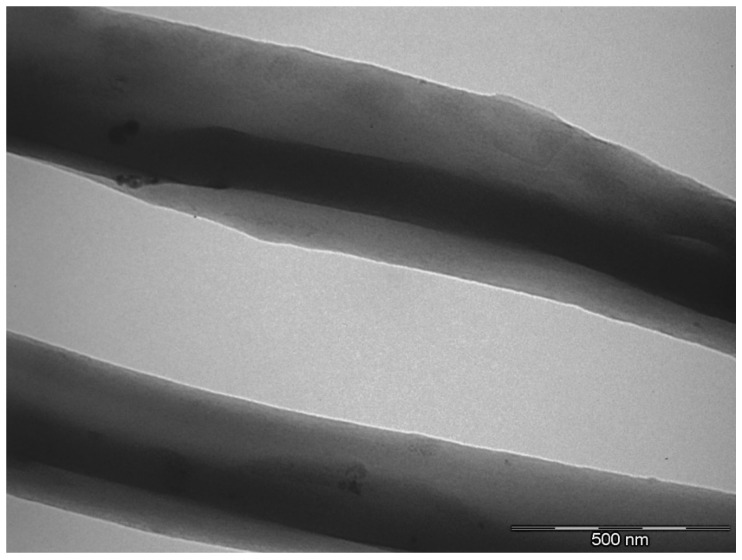
Transmission electron microscope (TEM) image of the electrospun poly(ethylene-*co*-vinyl alcohol) (EVOH) fibers containing graphene nanoplatelets (GNPs). Image corresponds to EVOH/GNP fibers at 0.5 wt.-%. Scale marker is 500 nm.

The presence and distribution of the GNPs in the electrospun EVOH fibers was also analyzed by TEM. Figure 4 displays a micrograph of the submicron EVOH fibers containing 0.5 wt.-% GNPs. In this image it can be observed that the GNPs were embedded and rolled up in the form of a continuous layer along the EVOH fiber axis. A good dispersion of the GNPs seems to be attained as a result of the favorable interfacial interaction between graphene and the EVOH matrix. Rolling and alignment of the GNPs can be ascribed to the inherent high resilience of the graphene layers combined with the extensional forces provided by the electrospinning process [4]. As a result, the GNPs were successfully incorporated by electrospinning into continuous submicron fibers of EVOH. The resultant roll-like morphology can be also advantageous to prevent restacking of the individual GNPs.

**Figure 5:**
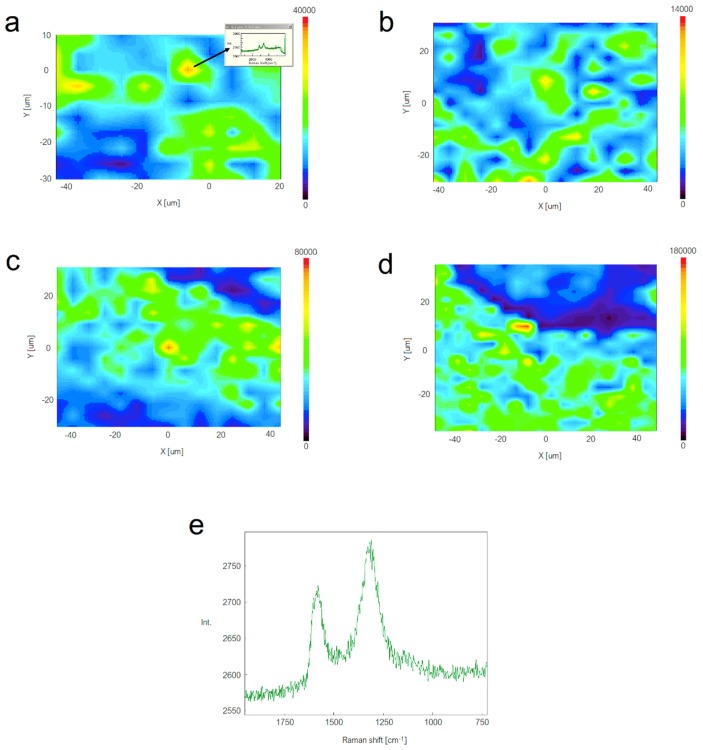
Raman spectroscopy contour plots of the electrospun poly(ethylene-*co*-vinyl alcohol) (EVOH) fibers containing graphene nanoplatelets (GNPs): (**a**) 0.1 wt.-%; (**b**) 0.5 wt.-%; (**c**) 1 wt.-%; (**d**) 2 wt.-%; (**e**) Typical non-confocal Raman spectrum of the sample EVOH/GNPs fibers at 2 wt.-%. As an example, Figure 5a shows as an inset the individual spectra that corresponds with the maximum signal of graphene in the image.

To further elucidate the GNPs dispersion in the submicron EVOH fibers, Raman analysis was carried out. Figure 5 shows typical Raman confocal images of the electrospun EVOH fiber mats reinforced with the different GNPs contents (Figure 5a–d) and also a typical non confocal spectrum of graphene taken in the electrospun mats (Figure 5e). The contour plots represent the added band areas of the two strong graphene bands across a defined sample area for the four nanocomposites. Thus, the more the yellow through to red color in the mapping, the stronger the presence of the GNPs. From the images, it seems that the dispersion was higher at lower nanofiller contents while the lowest dispersion, i.e., higher heterogeneity across the image, seemed to be for the sample with 2 wt.-% GNPs content, due to likely partial nanofiller agglomeration.

In relation to the individual spectra, the D-band at ~1316 cm^−1^ originates from the disordered carbon while the G-band, centered at ~1587 cm^−1^, associates with the ordered graphitic carbon [27]. In particular, the D-peak presence indicates the breakdown of translational symmetry in the lattice, which is attributed to either bulk defects in the basal plane or edge defects [28]. Table 1 includes the relative intensity ratio of D-band to G-band, i.e., ID/IG, which is habitually referred to as the “R-value”, for each electrospun fiber mat taken using a 40× objective in a non-confocal mode and, hence, averaging the graphene signal in the samples. This value can be used to quantitatively characterize the amount of structurally ordered graphite crystallites in carbonaceous materials. One can observe that the ID/IG ratio was kept constant at 0.98–0.99 for the electrospun EVOH fiber mats containing the GNPs in the 0.1–1 wt.-% range. This observation suggests that the GNPs were effectively dispersed as carbon nanofillers with ordered layers in the electrospun fibers. This is in good agreement with the recent findings by Li et al. [16], who additionally proposed that the embedded graphene particles played an important role in promoting graphitic crystallinity for PAN nanofibers. However, this value decreased to ~0.96 for the electrospun EVOH fibers filled with 2 wt.-% GNPs, which confirms that graphene was more agglomerated at the highest content. In addition, its higher separation in wavenumbers between the D-band and G-band (d_IG-ID_), also shown in Table 1, may also indicate a reduction in the number of layers present in the graphene tactoids, i.e., a lower degree of exfoliation [27].

### 3.3. Thermal Properties of EVOH/GNPs Fibers

**Figure 6:**
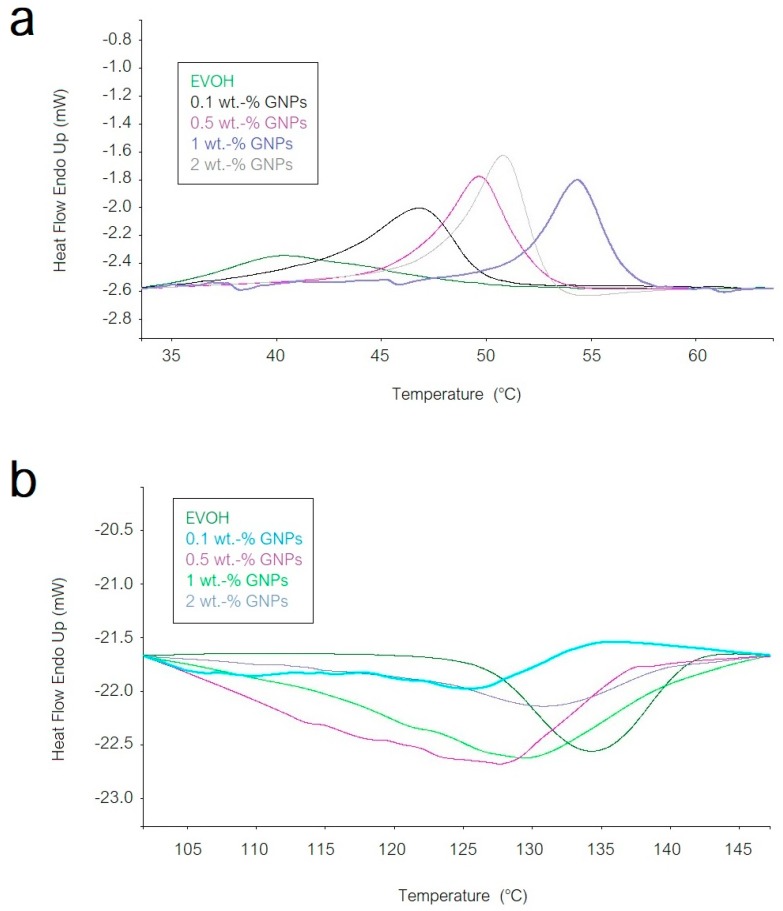
Differential scanning calorimetry (DSC) curves of the electrospun poly(ethylene-*co*-vinyl alcohol) (EVOH) fibers containing graphene nanoplatelets (GNPs): (**a**) First heating scan; (**b**) Cooling scan.

Figure 6 shows the DSC curves of the electrospun EVOH mats with the different GNPs contents. In Figure 6a it can be seen that the neat electrospun EVOH mat presented a relatively low glass transition temperature (T_g_), of around 40 °C, probably related to the presence of some remaining humidity in the fibers [29]. One can also observe that the T_g_ value increased with the GNPs content. Therefore, the presence of the carbonaceous nanoplatelets restricted the polymer chains motion. However, the highest T_g_ was observed at 54 °C, for the electrospun EVOH fibers containing 1 wt.-% GNPs, while the EVOH fibers with 2 wt.-% GNPs presented an intermediate T_g_ value of ~51 °C. This confirms that the GNPs agglomerated more strongly at their highest content, as supported by the above-described morphological and also chemical analyses. In addition, all samples presented a slight endothermic peak during glass transition, which can most likely be related to stress-relaxation mechanisms occurring in the vicinity of the T_g_. Other researchers have ascribed this effect to a physical-aging process. With aging of the material after processing, the polymer chains exhibit slow thermodynamic changes in the amorphous region to attain a lower-free energy state. As a result of this segmental mobility, the aged sample has smaller free volume and lower potential energy than the just processed one [30]. Therefore, when the materials are reheated, more energy is required to surpass the glass transition, which results in a small endothermic peak [31]. This molecular rearrangement has been previously described for EVOH films after pressure-assisted thermal processing [32].

In addition to the thermal variations during glass transition, all the submicron EVOH fibers did not present any melting peak during the first heating scan. This observation confirms that the electrospinning process led to a fully amorphous structure. A similar effect of chain mobility restriction in the EVOH fibers due to the presence of the GNPs can be observed in the cooling scan during crystallization from the melt, shown in Figure 6b. For instance, the electrospun EVOH fibers with 0.5 wt.-% GNPs presented a crystallization temperature (T_c_) of ~128 °C while the neat EVOH fibers showed a value of ~134 °C. This delay in crystallization supports the above-described statement that the embedded GNPs acted as an anti-nucleant agent, particularly at low contents due to their improved dispersion.

### 3.4. Dielectrical Performance and Electrical Conductivity of EVOH/GNPs Fibers

**Figure 7:**
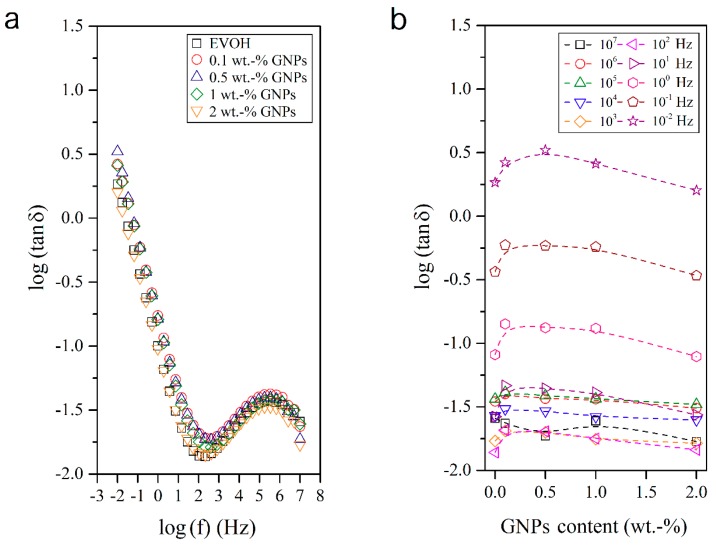
(**a**) Isothermal dielectric curves of the loss tangent (tan δ) of the electrospun poly(ethylene-*co*-vinyl alcohol) (EVOH) fibers containing graphene nanoplatelets (GNPs); (**b**) Influence of the GNPs content on tan δ for all frequency decades.

**Figure 8:**
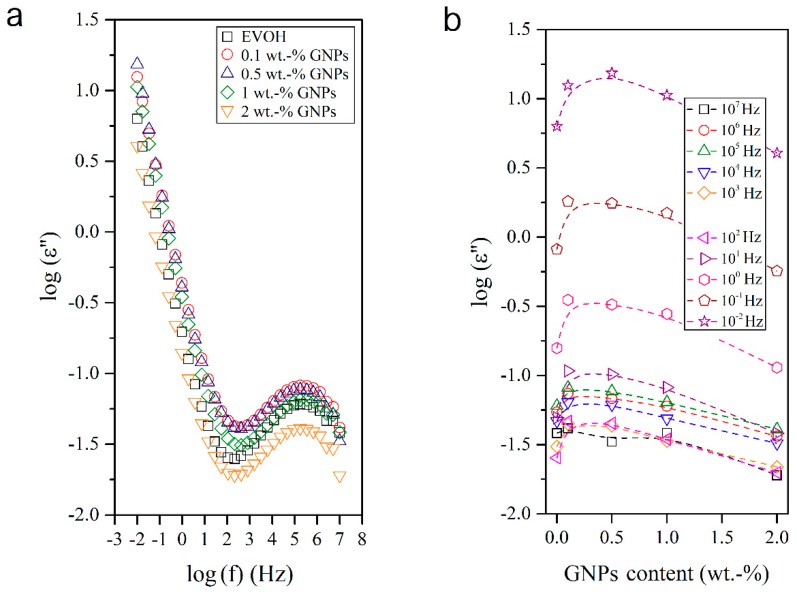
(**a**) Isothermal dielectric curves of the imaginary dielectric permeability (ε″) of the electrospun poly(ethylene-*co*-vinyl alcohol) (EVOH) fibers containing graphene nanoplatelets (GNPs); (**b**) Influence of the GNPs content on ε″ for all frequency decades.

**Figure 9:**
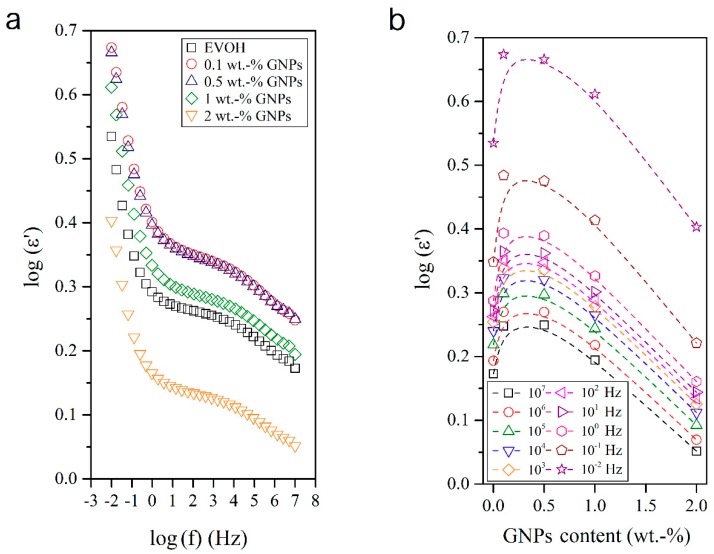
(**a**) Isothermal dielectric curves of the real dielectric permeability (ε′) of the electrospun poly(ethylene-*co*-vinyl alcohol) (EVOH) fibers containing graphene nanoplatelets (GNPs); (**b**) Influence of the GNPs content on ε′ for all frequency decades.

**Figure 10:**
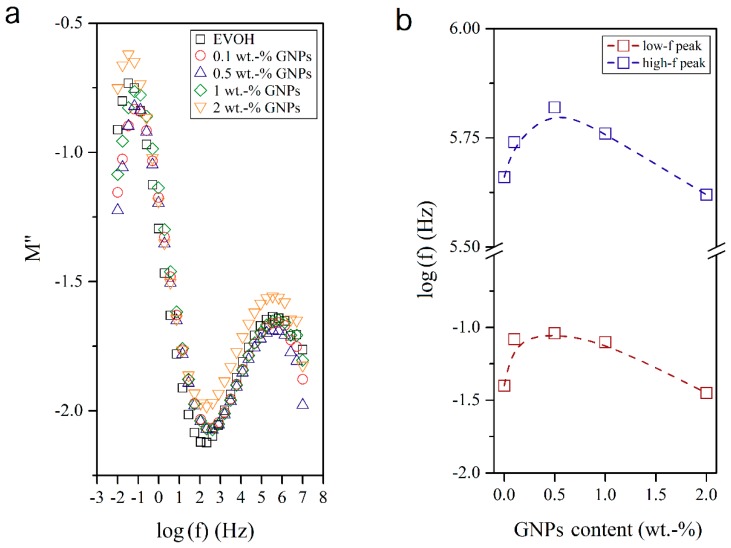
(**a**) Isothermal dielectric curves of the imaginary electric modulus (M″) of the electrospun poly(ethylene-*co*-vinyl alcohol) (EVOH) fibers containing graphene nanoplatelets (GNPs); (**b**) Influence of the GNPs content on the frequency (*f*).

The dielectric analysis of polymer materials and composites permits understanding the macromolecular relaxations [33,34,35,36] and is also a valuable approach for evaluating their conductivity and electric response. In this sense, Figure 7, Figure 8, Figure 9 and Figure 10 show the dielectric spectra of the EVOH and EVOH/GNP fibers in terms of ε′, ε″, tan δ, and M″, respectively, in all the frequency range at 25 °C. In Figure 7 and Figure 8 one can observe that, at low frequencies, tan δ and ε″ attained higher values though these values diminished rapidly with frequency. These results can be explained due to the fact that the alternation of the electric field was slow in the low frequency region, providing sufficient time for the permanent and induced dipoles to align themselves according to the applied field and, thus, leading to enhanced polarization. Broader dipolar polarization/relaxation processes were observed at higher frequencies, labelled as β-relaxation. This relaxation may be attributed to the local-mode relaxation in the crystalline regions of the copolymer and/or to the motion of their hydroxyl groups that could interact with each other. The neat EVOH fibers and EVOH/GNPs fibers exhibited almost identical values, with a slight increase when the GNPs were added. The maximum values were observed for a GNPs content of 0.5 wt.-%.

Figure 9 shows the frequency response of the isotherms of the electrospun submicron neat EVOH fibers and EVOH/GNPs composite fibers in terms of ε′. Three stages can be observed with the frequency increase. In brief, these involve a first decrease at low frequencies, followed by a plateau related to the copolymer relaxation processes, and a second decrease at high frequencies. Although all samples presented the same behavior, a non-linear slight increase was observed when the GNPs content was increased. Regardless of the magnitude of the frequency, the influence of the GNPs content showed similar profiles, with maximum values of dielectric permittivity around 0.5 wt.-% GNPs, which may indicate the most relevant polarization enhancement.

To ascertain the molecular origin of this effect, Figure 10 plots the variation of M″ in the neat EVOH fibers and EVOH/GNPs nanocomposite fibers. When the conductive effects were minimized, two peaks could be recorded, that is, one prominent peak at low frequencies and a broader one at high frequencies. These peaks have been described by other authors as the α- and β-relaxations, respectively [37]. On the one hand, the α-relaxation is ascribed to the main chain segmental motion and reflects a transition from the glassy to the rubbery state, related to the so-called T_g_. This is a large-scale cooperative process determined mainly by intermolecular interactions. One can observe that the presence of the GNPs shifted the α-relaxation peak to higher frequencies, showing a maximum of frequency for a content of 0.5 wt.-% GNPs in EVOH. It means that, at this particular composition, the segmental molecular mobility of the main chain was reduced and, therefore, the T_g_ value increased. However, when the GNPs composition was higher than 0.5 wt.-%, the frequency of the peak decreased. This further confirms that the nanoparticles may begin to agglomerate at higher contents, as supported by the above-described information during the morphological and thermal analyses. This fact highly influences the performance and especially the electrical properties of polymer nanocomposites [38]. On the other hand, the β-relaxation was attributed to the local-mode relaxation in the crystalline regions of the copolymer and/or to the motion of the hydroxyl groups. The β-relaxation peak also showed a maximum frequency value at 0.5 wt.-% GNPs.

**Figure 11:**
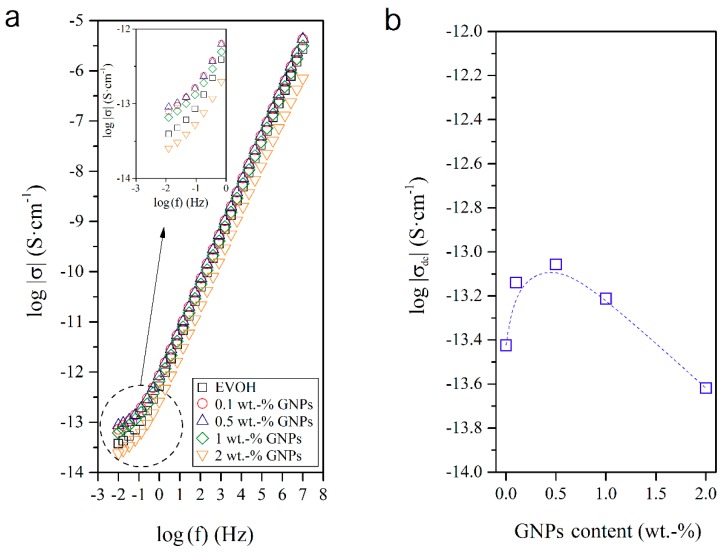
(**a**) Isothermal dielectric curves of the electrical conductivity (σ) of the electrospun poly(ethylene-*co*-vinyl alcohol) (EVOH) fibers containing graphene nanoplatelets (GNPs); (**b**) Influence of the GNPs content on the direct current electrical conductivity (σ_dc_).

Figure 11a shows the evolution of σ in the 10^−2^–10^7^ Hz frequency range. This property is typical of electrode polarization, associated with the accumulation of charges at the interfaces between the electrodes and the polymer sample, which increases with increasing frequency. This motion of charged carriers is spatially limited within their potential wells [39,40,41,42,43]. At lower frequencies, plateau regions occur, which can be associated with the free-charge transfer in the rubbery state and they are related to σ_dc_. However, at higher frequencies, the alternating current electrical conductivity (σ_ac_) occurs. The transition from linear non-frequency dependent σ_dc_ to the frequency dependent range of the σ_ac_ regions corresponds to the change in the mechanism of electrical conduction, which can be described by the movement of charges at long distances [40,44]. In Figure 11b one can observe that the dependence of σ_dc_ of the nanocomposite fibers on the GNPs content was not linear. Table 2 shows the values of σ_dc_ as a function of the GNPs content. The incorporation of low nanofiller contents sharply increased the conductivity of the electrospun EVOH fibers, up to a content of 0.5 wt.-% GNPs, with a percolation threshold relatively close to 0.1 wt.-% GNPs. The conductivity behavior of the nanocomposite fibers then changed, at very low GNPs contents, from an electrical insulator to a semiconductor material. However, at the highest GNPs contents tested, the σ_dc_ values significantly decreased. As commented above, this phenomenon can be related to a low dispersion of the carbonaceous nanoparticles, for which no conducting clusters or bridges were formed inside the electrospun submicron EVOH fibers.

### 3.5. Electrospun EVOH/GNPs Films

**Figure 12:**
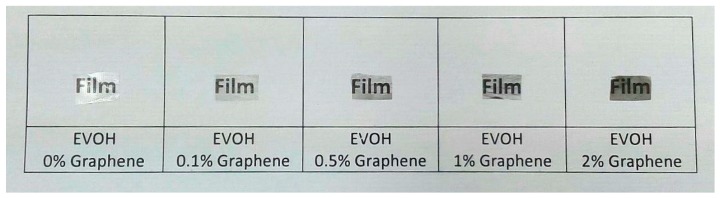
Electrospun films of poly(ethylene-*co*-vinyl alcohol) (EVOH) containing graphene nanoplatelets (GNPs) obtained by annealing.

Finally, Figure 12 shows the optical appearance of resultant EVOH/GNPs films obtained after thermal post-treatment at 158 °C, below the polymer’s T_m_, the so-called annealing, carried out on the electrospun fiber mats. Annealing applied on electrospun fibers mats results in continuous films that have significant potential for use in food packaging applications [45]. Simple naked eye examination of this image indicated that annealing produced continuous transparent films. This process has been recently ascribed to a compact packing rearrangement of the electrospun fibers by a phenomenon of fiber coalescence [46,47]. Another relevant observation is that, as the GNPs content increased, the resulting films became more opaque and developed a grey-like color, albeit contact transparency was preserved. This preliminary result indicates that the electrospun fibers mats can be turned into actual films, which will be the subject of further studies, since it may be advantageous for the application of developing labels or tags to have materials in a film format.

## 4. Conclusions

EVOH fibers containing GNPs were successfully prepared by electrospinning. To this end, the GNPs were firstly synthetized from graphite powder by oxidation to GO according to the Hummer’s method and, then, by reduction using NH_4_OH. The resultant flat-like graphene nanoparticles, the so-called GNPs, were solution electrospun with EVOH to obtain nanocomposite fibers with sizes in the submicron range. The embedded GNPs were rolled up in the form of continuous layers along the EVOH fiber axis. Both morphological and Raman analyses revealed that the best GNPs dispersion was obtained for the electrospun EVOH fiber mats containing 0.5 wt.-% GNPs, while graphene is likely to increasingly agglomerate as its concentration was increased. Thermal characterization indicated that the incorporated GNPs acted as an anti-nucleant agent for the EVOH molecules, particularly at low contents due to its improved dispersion and high interaction with the polymer matrix. Finally, the dielectric behavior of the nanocomposite fibers was studied in the frequency 10^−2^–10^7^ Hz range and as a function of the GNPs content. The dielectric constant was reduced with the frequency increase, in the whole range of frequencies, while it increased for GNPs contents up to 0.5 wt.-%, supporting the well dispersion of the nanoparticles at low loadings. In addition, the nanocomposite fibers presented high σ values in the 0.1–1 wt.-% GNPs range. The electrospun mat was, finally, thermally post-treated at 158 °C to produce continuous and contact transparent films. Applications of the resultant electrospun nanocomposite fiber mats and possibly also films in the intelligent packaging field as, for instance, smart labels or tags can be anticipated.

## Figures and Tables

**Table 1:** Distance in wavenumbers (d_IG-ID_) and relative intensity ratio of D-band to G-band (ID/IG) of the electrospun poly(ethylene-*co*-vinyl alcohol) (EVOH) fibers as a function of the graphene nanoplatelets (GNPs) content.

GNPs Content (wt.-%)	d_IG-ID_ (cm^−1^)	ID/IG
0.1	261	0.986
0.5	257	0.991
1	267	0.981
2	270	0.962

**Table 2:** Direct current electrical conductivity (σ_dc_) of the electrospun poly(ethylene-*co*-vinyl alcohol) (EVOH) fibers as a function of the graphene nanoplatelets (GNPs) content.

GNPs Content (wt.-%)	σ_dc_ (S cm^−1^) × 10^14^
0.0	3.77
0.1	7.25
0.5	8.78
1.0	6.14
2.0	2.41
